# The relationship between unhealthy food sales, socio-economic deprivation and childhood weight status: results of a cross-sectional study in England

**DOI:** 10.1186/s12966-016-0345-2

**Published:** 2016-02-15

**Authors:** Stephanie Howard Wilsher, Flo Harrison, Fred Yamoah, Andrew Fearne, Andy Jones

**Affiliations:** Norwich School of Medicine, University of East Anglia, Norwich, NR4 7TJ UK; Hertfordshire Business School, University of Hertford, Hatfield, AL10 9EU UK; Norwich Business School, University of East Anglia, Norwich, NR4 7TJ UK; UKCRC Centre for Diet and Activity Research (CEDAR), School of Clinical Medicine, University of Cambridge, Cambridge, CB2 0QQ UK

**Keywords:** Childhood, Weight status, Neighbourhood, Food purchases, Deprivation

## Abstract

**Background:**

Recent increases in obesity prevalence have led to research into the neighbourhood food environment. Research suggests that proximity and density of food outlets around the home is associated with childhood obesity prevalence, however, the evidence is inconclusive, and associations between food outlet locations and diet are less clear. The purpose of this study is to assess area level associations between sales of unhealthy foods in supermarkets and weight status of children.

**Methods:**

This study examined the association between weight status in children (4–5 year olds and 10–11 year olds) measured in the National Child Measurement Programme over three time points (2008/9, 2009/10, 2010/11) and annual sales of unhealthy foods (2012/3), as identified from a large supermarket chain. Geographical analysis was conducted to link store-based food sales for 537 stores with 6517 UK Census Areas. Unadjusted associations were examined with error-bar plots and linear regression was used to examine the relationship between the prevalence of overweight and obesity and sales of unhealthy food, while controlling for covariates known to predict weight status in children.

**Results:**

A statistically significant relationship was identified between the sales of unhealthy foods and the prevalence of overweight and obese children in both age groups (*p* < 0.01). Of the covariates, area deprivation was positively associated with weight status (*p* < 0.001). Non-white population (%) was negatively associated (*p* < 0.001) with overweight and obesity among Reception children, but positively associated with the other weight statuses (*p* < 0.001). A higher proportion of children in the same age group were associated with statistically significantly lower overweight and obesity prevalence in Reception (*p* <0.01) but not Year 6 children.

**Conclusions:**

The study provides novel findings linking supermarket food sales with the weight status of children. Food sales in geographically referenced supermarkets are a valuable source of data for research into the factors that influence the weight of the surrounding population. Future research could identify factors that might modify food shopping in supermarkets and use of purchasing data could be an effective way to measure the impact of healthy eating campaigns on the weight status of children over time.

## Background

The risk of becoming overweight or obese often starts in early childhood [1, 2] and in England, almost 23 % of children aged 4 and 5 years are overweight, of whom 9 % are obese. Prevalence of overweight and obesity are higher (34 % and 19 % respectively) for 11 to 15 year olds and are almost double in the most deprived areas compared to the least [3]. Excess weight in children is of concern due to the increased risk of a range of health issues including asthma, psychosocial morbidity, orthopaedic complications, cardiovascular problems and diabetes [4]. The cumulative effects on morbidity, disability and mortality continue into adulthood where around 64 % of individuals are overweight in England, of whom 25 % are obese [5]. This accumulating risk of obesity over the life course is attributable to many causes ranging from individual and family to social and environmental factors [6]. However individual factors alone cannot explain the rapid increase in obesity seen over a relatively short time, so the role of the environment has come under scrutiny [7].

The view is that in an obesogenic environment, where unhealthy foods high in fat and sugar are readily available and easily accessible, people become obese [8]. The food environment can be separated into macro-level and micro-level environments. Macro-level or the neighbourhood food environment covers proximity and density of food outlets and micro-level or consumer environment covers the food availability and accessibility within food outlets. Each level may represent a causal pathway to the consumption of an unhealthy diet and future obesity. However, researching the link between food environments, poor diet and obesity is challenging and has so far produced inconsistent results [9–11]. A review of the micro-level environment found mixed results in the association between the availability of healthy or unhealthy foods in stores, diet and weight status [11]. Healthy food was found to be cheaper in large stores, such as supermarkets compared to smaller stores, such as convenience stores and unhealthy foods were more common in the latter [11]. Regarding the macro environment, accessibility to supermarkets has been consistently associated with lower weight status. However, dietary behaviour does not seem to follow the same pattern [12]. For example, Jennings et al. [13] found that a greater number of food outlets selling healthy items (including supermarkets) within 1 km of the home were associated with lower weight status (BMI) in 9–10 year old English children, however no significant associations were found between supermarket availability and food intake.

Research to date has typically assessed the link between neighbourhood food environments and obesity or diet by quantifying the density and proximity of food outlets, often using small population samples covering limited geographic areas. Understanding how the food environment is actually used, by examining food sales within stores, is important for teasing out how the environment impacts individual behaviours that could increase the risk of being overweight or obese. With this in mind, this study uses data on sales of healthy and unhealthy foods at a leading national UK supermarket chain, and our analysis is based on the assumption that sales at any given supermarket are representative of those made by the population for whom that store is their nearest. Combining these data from stores in England with weight status measurement from the England-wide National Child Measurement Programme [14] undertaken amongst children aged 4–5 (Reception) and 10–11 (Year 6), we test the hypothesis that increased local purchasing of unhealthy foods is associated with a higher prevalence of childhood overweight and obesity in an area.

## Methods

### Study population and anthropometric measurements

The English National Child Measurement Program (NCMP) was developed to monitor rates of overweight and obesity in English primary school children. The program aims to record the height and weight of all children in Reception (4–5 years old) and Year 6 (10–11 years old) in England using standardised anthropometric procedures by trained staff. Height is measured to the nearest whole or half centimetre using a floor mounted stadiometer with the head in the Frankfort Plane. Weight is measured in kilograms to one decimal place using class III scales. Children are asked to remove shoes and outdoor clothing for both measurements and are weighed in normal light indoor clothing [15]. The resultant data are available for around one million children each year, averaging 92 % of eligible children in England [14]. The data have been collected annually since 2005 and are available aggregated to a variety of geographic units. For this study, the prevalence of overweight and obesity were obtained for all Middle Super Output Areas (MSOAs) in England from the measurement years 2008/09, 2009/10, and 2010/11. Prevalence was averaged across three time points in each MSOA to reduce annual variability of participation in the measurements in the relatively small child populations in each geographical unit for which data were available. MSOAs are a unit of UK Census geography designed for small-area analyses [16]. The 6781 MSOAs in England have an average of 7500 inhabitants and contain around 190 children in each of the NCMP age groups. Within the NCMP data, overweight and obesity were defined as a body mass index (BMI) greater than or equal to the 85^th^ and 95^th^ percentile of the UK90 BMI reference respectively [1, 17].

### Food sales data

Data on volume of food sales was obtained from a large supermarket chain, comprising 538 stores. The data comprised food purchased from nine food categories during a 52-week period covering mid-August 2012 to mid-August 2013, based on a 10 % sample of the retailer’s eighteen million loyalty card holders. The categories included: fresh and frozen fruit and vegetables, cakes, biscuits, savoury pies, savoury snacks, and sweetened drinks. We chose these foods as they can be classified relatively unambiguously as either “healthy” or “unhealthy”. Fruit and vegetables are synonymous with a healthy diet and recommended by the Department of Health, NHS, whereas foods such as cakes, biscuits, savoury pies and snacks, and sweetened drinks are considered unhealthy due to the high fat and/or sugar content and consumption should be limited [14]. Sales of these unhealthy foods are likely to be for consumption by children. The healthy foods did not include tinned and dried fruit and vegetables due to lack of available data. Other healthy foods such as dried pulses, seeds and nuts were not included as these may not be regularly consumed by children.

Volume of sales data were aggregated to the store level for supermarkets of the chain. Fruit and vegetables sold loose were excluded from our analysis as only the number of transactions, rather than the units purchased, are recorded making them not comparable with pre-packed sales. Sales of loose fruit and vegetables are relatively low however, accounting for 18.5 % of all fruit and vegetable transactions at the supermarket chain.

As the absolute volume of sales varied significantly between stores we used a composite measure – the sale of unhealthy foods as a percentage of total sales for the nine food categories (Unhealthy Foods Sales Percentage -UFSP) for each store and divided them into quartiles.

A geographic analysis was undertaken in order to link the store-based food sales data to MSOAs. The postcodes (zip-codes) of supermarket stores were geocoded in a Geographical Information System (GIS) (ArcGIS 10.1 (ESRI Inc, Redlands, CA, USA)) based on postcode locations. A proximal region was then delineated around each store based on Euclidian straight-line distance. The proximal regions define the area around a supermarket for which that store is the nearest, so are by definition space-filling and non-overlapping. The creation of these regions, also known as Theissen polygons, is a standard procedure in geographic analysis [18]. In this study, the proximal regions estimate the area from which each store draws its customers, based on the assumption that shoppers use the nearest store. Each MSOA was linked to a supermarket based on the proximal region its geographic centre fell within.

### Covariates

In order to adjust our analyses for known area-level correlates of childhood obesity and its behavioural determinants we obtained a range of measures for each MSOA from national data agencies. The Income Deprivation Children Index (IDACI) measures the proportion of children aged up to 16 years living in low income households and was obtained from the UK data service based on 2010 data [19]. Measures of population ethnicity (% non-white), and age structure; % aged under 7 years for models of weight status in Reception children, and % aged 10–14 for Year 6 models, were obtained from the UK 2001 census [20]. The 2001 Census was used by the National Obesity Observatory for the aggregation of the NCMP obesity data for 2008–2011, and allowed access to demographic data for the same geographic boundaries. Both deprivation and non-white ethnicity have been associated with increased risk of obesity [21], while the number of similar age children in each MSOA is an indicator of possible social networks thought to be important for both diet and physical activity behaviours [22].

### Statistical analysis

Unadjusted associations between UFSP quartiles and weight status outcomes, the percentage of obese and percentage overweight and obese children in Reception and Year 6 in each MSOA, were examined using error-bar plots. Linear regression was used to examine the relationship between the UFSP and the weight status outcomes while controlling for covariates. In order to examine adjusted trends, the models produced were used to predict weight status outcomes for each UFSP quartile at the mean values of other covariates. All statistical analyses were performed using SPSS version 22 (IBM Corp, Armonk, NY, USA).

## Results

In total 209 (3.1 %) of the MSOAs had missing data for Reception obese and overweight and 62 (0.9 %) missing for the Year 6 obese and overweight due to data suppression as a result of low numbers [14]. There was no store on the Isles of Scilly and sales data for a superstore in Gateshead, Tyne & Wear were missing, resulting in the removal of a further 40 MSOAs from the analyses and giving a final sample of 6517 MSOAs (96 % of all English MSOAs). Sales data was available for 537 (99.8 %) supermarkets in England. Their proximal areas range in size from 11 km^2^ to 2695 km^2^ (mean 246 km^2^, SD 278 km^2^), and contain an average of 12.6 MSOA centroids (SD 8.7). Summary statistics describing the data for the MSOAs included in these analyses are presented in Table 1. The lowest quartile of UFSP represents the lowest sales of unhealthy foods in relation to total sales for the nine food groups. In terms of food sales, units sold of fresh fruit and vegetables were orders of magnitude higher than their frozen counterparts, and sweetened drinks were the most sold of the unhealthy food types included.

**Table 1:** Descriptive statistics of data generated for English Middle Super Output Areas (MSOA)

	Mean (Standard deviation), or Median; 25th centile - 75th centile
Weight status	
% Reception children overweight or obese	23.5 (4.4)
% Reception children obese	9.4 (2.9)
% Year 6 children overweight or obese	34.5 (5.9)
% Year 6 children obese	18.7 (5.0)
Food sales	
Biscuits	68520; 39110 - 107710
Cakes	172280; 99710 - 271980
Crisps	120580; 72130 - 191150
Fresh Fruit	937490; 560120 - 1392990
Fresh Vegetables	733180; 410660 - 1113200
Frozen Fruit	6840; 3857.5 - 11330
Frozen Vegetables	80640; 49080 - 130380
Sweetened drinks	358390; 217540 - 561210
Pies	305450; 184470 - 467160
Demographic co-variates	
Income deprivation affecting children (IDACI)	0.17; 0.10 - 0.30
% MSOA population aged under 7 years	9.8 (2.0)
% MSOA population aged 10–14 years	6.6 (1.2)
% MSOA population of non-white ethnicity	1.4; 0.5 - 5.8

Note- Weight status - average prevalence across 2008/9, 2009/10, 2010/11), Food sales represent units sold 2012/13, demographic details for MSOA are from the 2001 UK Census, and IDACI data were collected in 2010

**Fig. 1:**
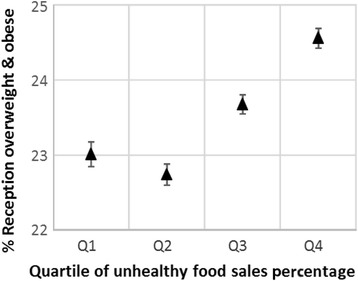
Unadjusted mean prevalence (and 95 % confidence intervals) of overweight and obesity in Reception children by quartile of unhealthy food sales percentage (UFSP). Note – the lowest quartile of UFSP represents the lowest sales of unhealthy foods in relation to total sales for the nine food categories

**Fig. 2:**
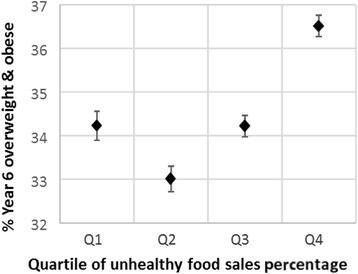
Unadjusted mean prevalence (and 95 % confidence intervals) of overweight and obesity in Year 6 children by quartile of unhealthy food sales percentage (UFSP). Note – the lowest quartile of UFSP represents lower sales of unhealthy foods in relation to total sales for the nine food categories

Before adjustment, the percentage of children in each MSOA who were overweight and obese was statistically significantly associated with UFSP for children in both Reception (Fig. 1) and Year 6 (Fig. 2), with tests for trend being *p* < 0.01. The models for obesity alone were very similar and not shown here. However, this was not a simple linear relationship, with the mean prevalence dropping between UFSP quartiles one and two before increasing again through quartiles three and four. For all four outcomes, mean prevalence was statistically significantly higher in UFSP quartile four than quartile one.

Results from the regression models used to adjust for potential confounders are shown in Table 2. For all four outcomes, the UFSP remained a statistically significant predictor of weight status with a statistically significant trend (*p* < 0.01). Of the covariates included in these models, area deprivation was consistently positively and significantly (*p* < 0.001) associated with both overweight and obesity prevalence combined, and obesity alone. The percentage of the population that was non-white was negatively but significantly (*p* < 0.001) associated with overweight and obesity among Reception children, but showed a positive significant (*p* <0.001) association with the other three outcomes. A higher proportion of children in the same age group were associated with statistically significantly lower overweight and obesity prevalence in Reception but only for overweight and obesity combined in Year 6 children.

**Table 2:** Results from regression models of weight status outcomes

	% Overweight or Obese					% Obese				
		95 % CI					95 % CI			
	B	lower	upper	p		B	lower	upper	p	
Reception (children aged 4–5 years)										
Quartile of % UFSP^a^										
1 (lowest)	-ref-	-	-	-		-ref-	-	-	-	
2	0.479	0.216	0.743	**<0.001**		0.235	0.068	0.401	**0.006**	
3	0.965	0.698	1.232	**<0.001**		0.533	0.364	0.702	**<0.001**	
4 (highest)	1.170	0.902	1.439	**<0.001**	*	0.804	0.635	0.974	**<0.001**	*
Area deprivation score	17.438	16.640	18.236	**<0.001**		11.782	11.278	12.286	**<0.001**	
% of MSOA population non-white	−0.019	−0.028	−0.011	**<0.001**		0.016	0.010	0.021	**<0.001**	
% of MSOA population under 7	−0.090	−0.144	−0.036	**0.001**		−0.050	−0.084	−0.016	**0.004**	
Year 6 (Children aged 10–11 years)										
Quartile of % unhealthy food sales^a^										
1 (lowest)	-ref-	-	-	-		-ref-	-	-	-	
2	0.438	0.123	0.753	**0.006**		0.460	0.203	0.718	**<0.001**	
3	1.299	0.979	1.620	**<0.001**		1.093	0.831	1.355	**<0.001**	
4 (highest)	2.654	2.329	2.980	**<0.001**	*	2.386	2.120	2.652	**<0.001**	*
Area deprivation score	22.288	21.375	23.201	**<0.001**		21.377	20.630	22.124	**<0.001**	
% of MSOA population non-white	0.072	0.062	0.081	**<0.001**		0.038	0.030	0.045	**<0.001**	
% of MSOA population 10–14 years	−0.112	−0.210	−0.014	**0.025**		−0.079	−0.160	0.001	*0.053*	

^a^Reference = Quartile 1, the lowest % unhealthy food sales percentage (UFSP). For p, **bold** font indicates *p* < 0.05, and *italic* font indicates statistical non-significance (*p* > 0.05). *test for trend across quartiles statistically significant (*p* < 0.05)

**Fig. 3:**
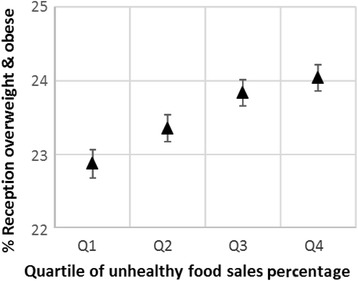
Adjusted^1^ mean prevalence (and 95 % confidence intervals) of overweight and obesity in Reception children by quartile of unhealthy food sales percentage (UFSP). Note – the lowest quartile of UFSP represents lower sales of unhealthy foods in relation to total sales for the nine food categories

**Fig. 4:**
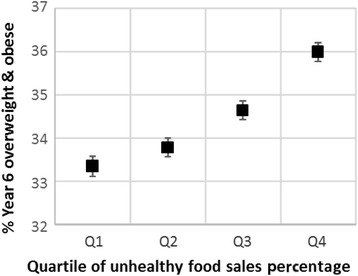
Adjusted^1^ mean prevalence (and 95 % confidence intervals) of overweight and obesity in Year 6 children by quartile of unhealthy food sales percentage (UFSP). Note – the lowest quartile of UFSP represents lower sales of unhealthy foods in relation to total sales for the nine food categories

Adjusted means and confidence intervals based on the models for overweight and obese are shown in Figs. 3 and 4. The models for obesity alone were very similar and are not shown here. For each outcome, the difference in obesity prevalence between top and bottom UFSP quartiles is somewhat attenuated compared to the trends in Figs. 1 and 2, but the relationship between weight status and the UFSP is more clearly linear. As with the unadjusted values, the effect size for Year 6 children was greater than for Reception children for both outcome measures. For overweight and obesity the difference in prevalence between quartile one and quartile four was 1.2 % for Reception, and 2.7 % for Year 6, while for obesity the differences were 0.8 % and 2.4 % respectively (all *p* < 0.01).

## Discussion

Our results show a clear association between supermarket sales of unhealthy foods as a percentage of overall sales (UFSP) and the prevalence of overweight and obese children among both Reception and Year 6 age groups. This association was attenuated somewhat after adjustment for potential confounders, but remained statistically significant. We found a larger effect size for Year 6 compared to Reception children, and their likelihood of being overweight or obese increased with higher sales of unhealthy foods.

The difference in prevalence of elevated weight status between Reception and Year 6, and the stronger association between the UFSP and weight status rates among Year 6 children suggests incremental build-up of environmental drivers that increase weight status. Older children have had longer exposure to the food environment and may have more autonomy to buy foods. Indeed, food outlets around schools [23, 24] and near the homes of children are associated with higher weight status in children [13].

Supermarkets are often categorised as a healthy food outlet [13, 25, 26] with their presence thought to increase the local availability of healthy foods, specifically fruit and vegetables [10]. Large supermarket chains such as the one included in this study have been shown to provide healthier in-store environments than other supermarket types (e.g. discount stores) [27]. However, there is growing realisation that they also provide access to a large number of unhealthy foods, the purchasing of which may be patterned by age [28] and socioeconomic status [29]. Our results show that the sales of unhealthy foods relative to total sales for the nine food groups at supermarkets is associated with increased prevalence of overweight and obesity, supporting the need for more careful consideration of the classification of supermarkets as health or unhealthy outlets when attempting to characterise the local food environment. Our findings highlight the potential limitations of mapping food outlets without understanding food purchasing behaviour, which this study begins to unravel.

We found that including area deprivation in our adjusted model attenuated associations between purchases of unhealthy foods and children’s weight status. Area deprivation may be associated with weight status as outlets selling unhealthy foods may be clustered in more deprived areas, although the evidence to support this is equivocal [30]. Area level deprivation may also be used as a proxy measure of the socio-economic status of individuals, and so associations seen between area measures may reflect the differing behaviours of residents, whereby individuals of lower socioeconomic status are likely to live in more deprived areas, and to have different food purchasing habits. Ransley et al. [29] analysed dietary fat and energy intake using supermarket till receipts in a sample of Tesco customers. They found that food with higher than recommended levels of fat and energy were more likely to be purchased by those with lower socioeconomic status. Results of another study using similar groups of healthy and unhealthy foods and Tesco loyalty card data of customers segmented by home tenure, marital status and affluence, found poorer families, single parents and council tenants, consistently purchased more unhealthy foods and generally less healthy foods that other demographic groups [31]. For such households, income may influence dietary behaviour [32], but will undoubtedly interact with many individual psychological and social factors [33, 34].

The strengths of the study include the large sample size, covering the whole of England, and the use of objective measures of both weight status and food sales. To our knowledge, no other study has used unhealthy food sales at individual store level and linked it to overweight and obesity prevalence in small geographical areas in England. However, Limitations must also be acknowledged. The weight status and sales data used for this analyses are from different time periods. Weight status measures were conducted from 2008–2011, and the sales data from 2012–2013. However, we believe that these data give adequate measures of the geographic distribution of both obesity and food sales at a national scale over that 5-year period. The use of a weight status measure averaged across three measurement cycles would have improved the stability of the geographic distribution, as would the aggregation of sale into broad food groups (healthy vs unhealthy). Obese children were more likely to opt out of the measurement scheme, suggesting the associations found in our analyses may have been stronger if all the children were measured [14]. The tenet of our analyses is based on the assumption that sales at any given supermarket are representative of food purchases made by the population for whom that store is their nearest. We believe that this is reasonable as research suggests that convenience and location are important considerations in store choice, especially for those with time pressures [35–37]. Although the linkage between MSOA and supermarket proximal area is determined by the home location, the scale of the proximal areas means that they cover areas large enough to include both home and work location. The mean distance from MSOA centroid to supermarket was 4.7 km, and in England 49.2 % of the working population report travelling less than 5 km to get to work [38]. The data relates to one supermarket chain, albeit a large national one. It is possible, but unlikely, that the purchasing behaviour of shoppers at this supermarket chain is not representative of supermarket shopping behaviour in general. The study was cross-sectional and so causality cannot be determined and the findings may equally reflect that customers with already higher weight status actively seek out the unhealthy foods.

## Conclusions

Our findings show a clear association between the sales percentage of unhealthy foods to total sales for the nine food categories (UFSP) within a supermarket and the prevalence of overweight and obesity among both Reception and Year 6 children in the locality. The association was stronger for older children; supporting the notion that cumulative exposure to the food environment contributes to weight status. Results also suggest that purchasing behaviour within food outlets, as well as outlet location should be considered in future work on food environments. In addition the findings highlight the problem of classifying food outlets for analysis as supermarkets, commonly used as a proxy for easy access to healthy fruit and vegetables, also provide easy access to unhealthy foods. Using sales data, including the sales of unhealthy foods relative to healthy foods, could be a highly effective way to measure the success of healthy eating campaigns over periods of time.

## Abbreviations

IDACI: Income Deprivation Children Index
MSOA(s): Middle Super Output Area(s)
NCMP: National Child Measurement Program
UFSP: Unhealthy food sales percentage
